# A noteworthy clue for diagnosing non-ST-elevation myocardial infarction by computed tomography without ECG synchronization: a case report

**DOI:** 10.1186/s12872-020-01512-2

**Published:** 2020-05-25

**Authors:** Tetsuya Nomura, Satoshi Tasaka, Kenshi Ono, Yu Sakaue, Naotoshi Wada, Natsuya Keira, Tetsuya Tatsumi

**Affiliations:** Department of Cardiovascular Medicine, Kyoto Chubu Medical Center, 25, Yagi-Ueno, Yagi-cho, Nantan City, Kyoto, 629-0197 Japan

**Keywords:** Non-ST-elevation acute coronary syndrome, Contrast-enhanced computed tomography, Electrocardiography synchronization, Myocardial perfusion, Percutaneous coronary intervention

## Abstract

**Background:**

Although timely coronary intervention can result in markedly improved clinical outcomes of patients with acute coronary syndrome (ACS), non-ST-elevation (NSTE)-ACS is sometimes difficult to accurately diagnose.

**Case presentation:**

A 52-year-old woman complained of acute chest pain with sudden onset. Both electrocardiography (ECG) and echocardiography showed normal results, and we urgently needed to make a differential diagnosis among critical illnesses such as acute coronary syndrome and nonischemic cardiovascular life-threatening diseases. Contrast-enhanced computed tomography (CT) without ECG synchronization showed evidence of neither aortic dissection nor pulmonary embolism, but regionally reduced contrast enhancement in the posterior myocardium, which were suggestive of myocardial ischemia. Emergency coronary angiography demonstrated severe stenosis of the left circumflex artery, and we achieved favorable revascularization with drug-eluting stent deployment.

**Conclusions:**

We diagnosed a patient with NSTE-ACS in whom contrast-enhanced CT without ECG synchronization was effective for visualization of reduced myocardial perfusion, suggesting ischemic heart disease.

## Background

When we encounter patients with acute chest pain, it is essential to make a prompt differential diagnosis among critical illnesses such as acute coronary syndrome (ACS), acute aortic dissection, and acute pulmonary embolism (PE). Though ACS is associated with relatively high morbidity and mortality, it is well-known that timely reperfusion therapy can result in markedly improved clinical outcomes [1]. However, non-ST-elevation-ACS (NSTE-ACS) is sometimes difficult to accurately diagnose, which often means that the opportunity to perform reperfusion therapy in timely manner is missed. A normal electrocardiography (ECG) does not exclude NSTE-ACS and occurs in 1 to 6% of those patients [2]. A normal ECG may be associated with left circumflex (LCX) or right coronary artery lesions, which can be electrically silent.

## Case presentation

**Fig. 1:**
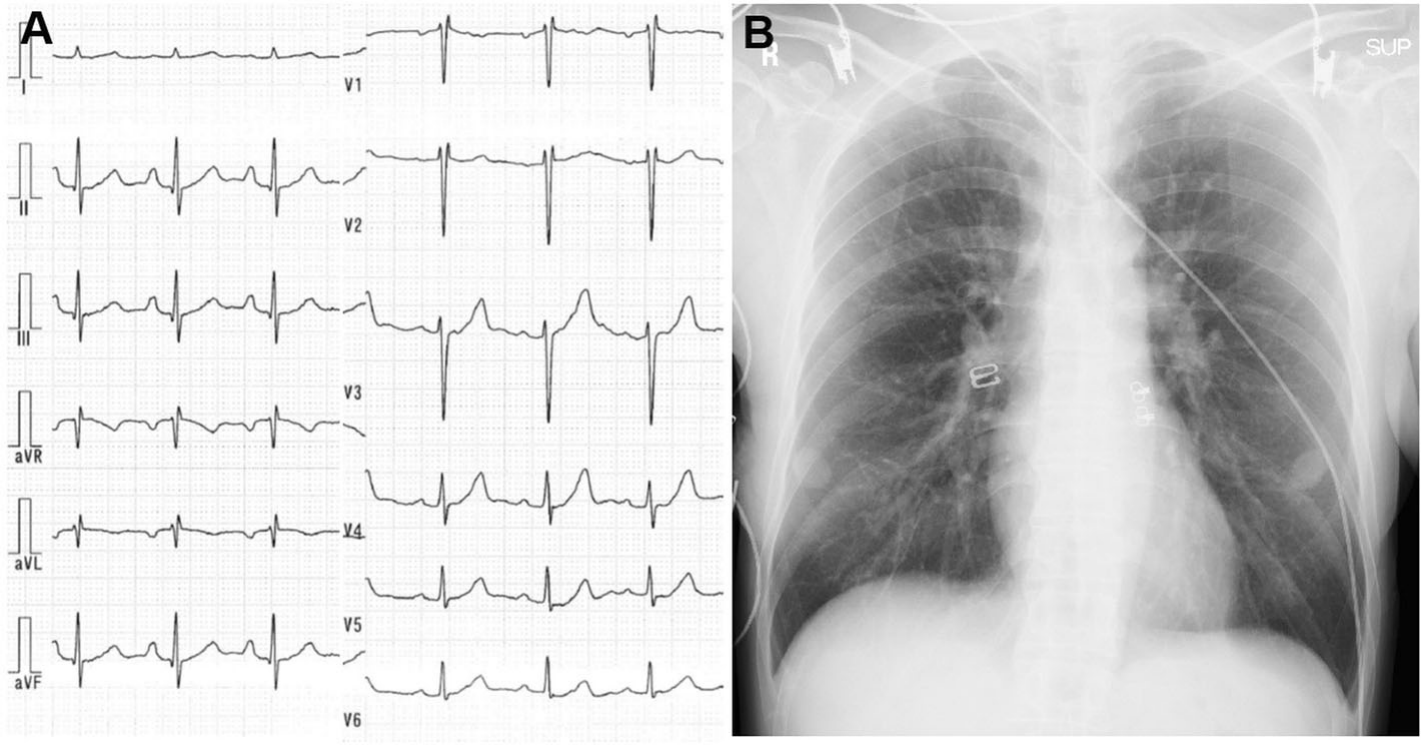
**a** A twelve-lead ECG showing no significant sign of cardiac ischemia. **b** A chest X-ray showing a normal cardiothoracic rate and absence of pulmonary congestion

**Fig. 2:**
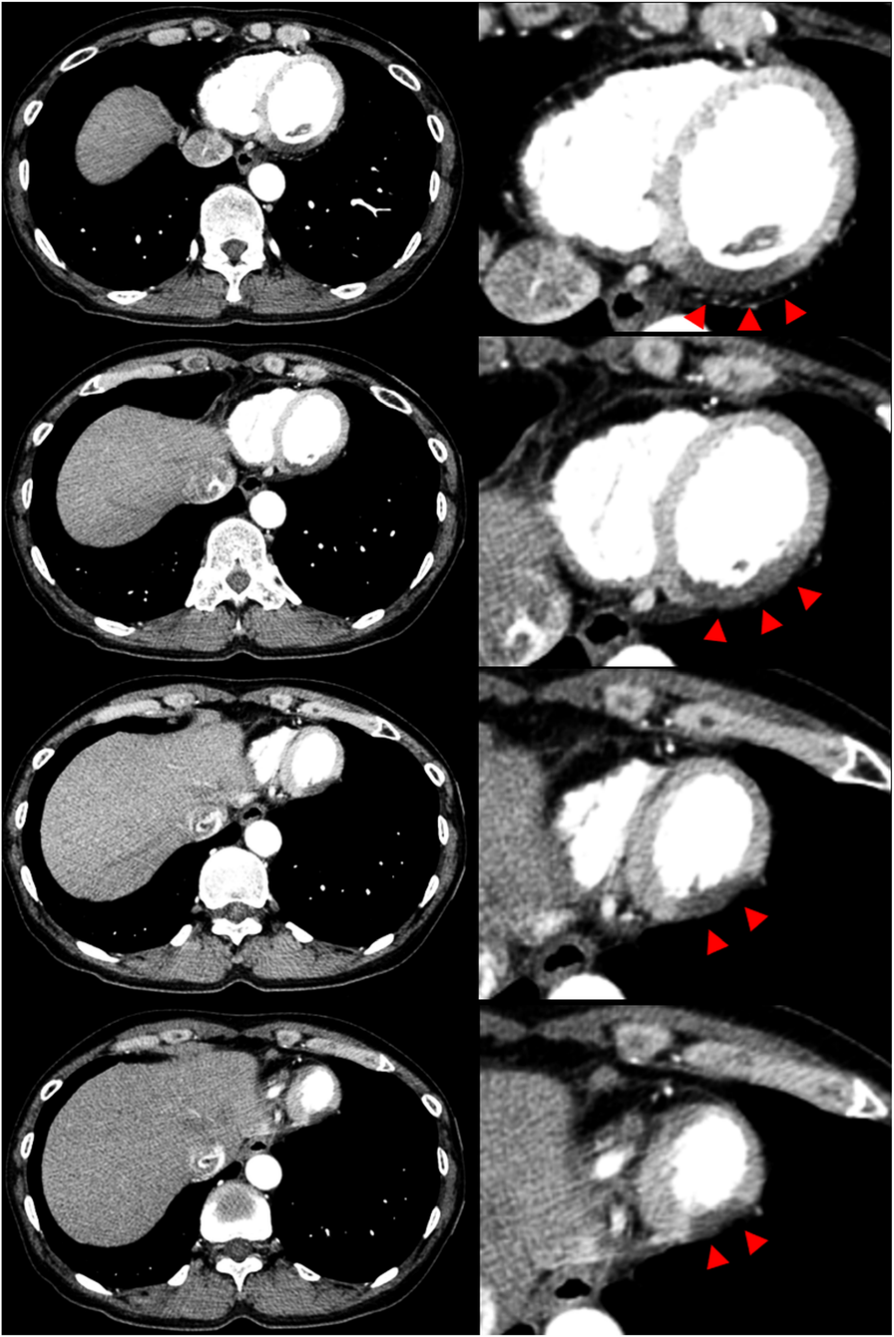
Axial images of contrast-enhanced CT without ECG synchronization demonstrating reduced myocardial perfusion in the posterior walls (left panels). The magnified images of the heart corresponding to each of the left sided image (right panels). Arrowheads showing the area of reduced myocardial perfusion

**Fig. 3:**
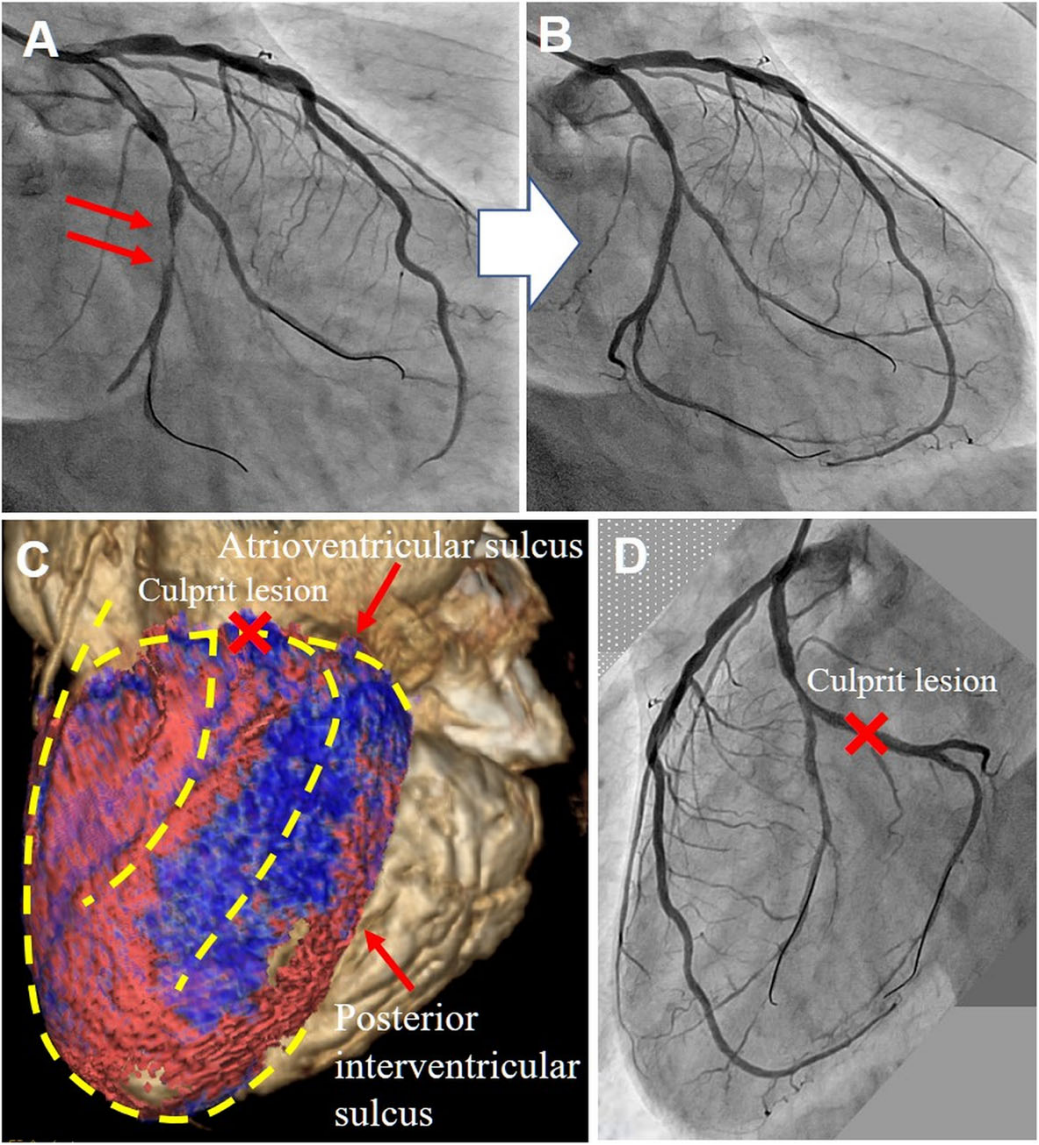
**a** Severe stenosis of the LCX artery on emergency CAG (Arrows). **b** Favorable arterial dilatation after coronary intervention. **c** A reconstructed volume rendering image showing an area of reduced contrast enhancement (blue area) and the supposed left coronary artery course (yellow dot lines). **d** Angiographical image of the left coronary artery viewed from the left side

A 52-year-old woman was admitted to our hospital complaining of persistent chest symptom with sudden onset. The symptom was an anterior chest squeezing with cold sweat and slight difficulty in breathing, which did not show symptom migration. She was a current smoker, but had no other cardiovascular risk factors. Her blood pressure was 112/72 mmHg, and pulse was 79/min and regular. Her body mass index was 22.6. No particular finding was noted on physical examination, and laboratory data showed a positive sign of Troponin T and increased level of leucocytes (13,180/μL) (Table 1). No significant sign of cardiac ischemia was noted on twelve-lead ECG (Fig. 1a). A chest X-ray showed a normal cardiothoracic ratio (42.0%) and absence of pulmonary congestion (Fig. 1b). The left ventricular wall motion was within normal limits and neither apparent valvular disorder nor a finding of pressure overload on the right side of the heart was seen on ultrasound echocardiography (UCG). We performed contrast-enhanced computed tomography (CT) without ECG synchronization, and detected evidence of neither aortic dissection nor PE. On the other hand, regionally reduced contrast enhancement in the posterior myocardium was clearly shown on axial CT images (Fig. 2). This CT image analysis was assessed using a window width and level of 300/50 Hounsfield units and a slice thickness of 1.25-mm. These findings were highly suggestive of myocardial ischemia. Then, we promptly conducted coronary angiography, and confirmed the presence of a severe stenotic lesion accompanied by coronary flow delay in the mid-portion of the LCX artery (Fig. 3a). We deployed a drug-eluting stent there, and achieved favorable vascular dilatation and coronary blood flow (Fig. 3b). Creatine phosphokinase was increased up to 377 IU/L after coronary intervention. Her post-procedural clinical course was stable and she was safely discharged 7 days after admission.

**Table 1:** Laboratory data on admission

**N**	**141 mmol/dL**	**TCH**	**195 mg/dL**
**K**	**3.7 mmol/dL**	**TG**	**91 mg/dL**
**Cl**	**107 mmol/dL**	**HDL-C**	**40.4 mg/dL**
**BUN**	**10.9 mg/dL**	**LDL-C**	**139.7 mg/dL**
**Cre**	**0.59 mg/dL**		
**Amy**	**64 IU/L**	**BNP**	**17.2 pg/mL**
**CPK**	**59 IU/L**	**Trop T**	**(+)**
**AST**	**15 IU/L**		
**ALT**	**10 IU/L**	**WBC**	**13,180 /μL**
**LDH**	**222 IU/L**	**RBC**	**465x10**^**4**^**/μL**
**ALP**	**346 IU/L**	**Hgb**	**14.7 g/dL**
**T-Bil**	**0.37 mg/dL**	**Hct**	**43.3 %**
**CRP**	**0.1 mg/dL**	**PLT**	**25.7x10**^**4**^**/μL**
**BS**	**121 mg/dL**		
**HbA1c**	**6.0 %**	**PT**	**107 %**
**TP**	**5.8 g/Dl**	**APTT**	**29.6 sec**
**Alb**	**3.7 g/dL**	**D-dimer**	**0.5 μg/mL**

*Amy* amylase, *AST* aspartate aminotransferase, *Alb* albumin, *ALP* alkaline phosphatase, *ALT* alanine aminotransferase, *APTT* activated partial thromboplastin time, *BNP* brain natriuretic peptide, *BS* blood sugar, *BUN* blood urea nitrogen, *CPK* creatine phosphokinase, *Cre* serum creatinine, *CRP* C-reactive protein, *HDL-C* high density lipoprotein-cholesterol, *LDL-C* low density lipoprotein-cholesterol, *Hct* hematocrit, *HbA1c* hemoglobin A1c, *Hgb* hemoglobin, *LDH* lactate dehydrogenase, *PLT* platelet count, *PT* prothrombin time, *RBC* red blood cell count, *T-Bil* total bilirubin, *TCH* total cholesterol, *TG* triglyceride, *TP* total protein, *WBC* white blood cell count

## Discussion and conclusions

ACS, including NSTE-ACS and ST elevation myocardial infarction, is usually due to an acute thrombotic occlusion of coronary arteries, associated with atheromatous plaque rupture or erosion. ACS is associated with a markedly impaired prognosis and requires immediate treatment [3]. The 2014 AHA/ACC guidelines state that the goal of immediate treatment of patients with NSTE-ACS is to provide relief from ischemia and prevent the recurrence of adverse ischemic events [4]. Differential diagnosis of NSTE-ACS includes nonischemic cardiovascular causes of chest symptoms (e.g. aortic dissection, expanding aortic aneurysm, pericarditis, pulmonary embolism), which are usually life-threatening illnesses. Therefore, establishing an accurate diagnosis of acute chest symptoms without delay is of marked importance to start treatment promptly. In NSTE-ACS, ECG change suggestive of ischemia such as ST depression, transient ST-elevation, or new T-wave inversion is often present and associated with elevated cardiac troponin. However, a normal ECG does not exclude NSTE-ACS and occurs in 1 to 6% of those patients [2]. A normal ECG may be associated with LCX or right coronary artery lesions, which can be electrically silent. For these reasons, NSTE-ACS is sometimes difficult to accurately diagnose, which often means that the opportunity to perform reperfusion therapy in a timely fashion is missed. There may be marked potential, greater for NSTE-ACS than STEMI, to improve outcomes through earlier and more accurate diagnosis of ACS [5].

Coronary CT has become more popular due to marked advancements in its performance, and it is not exaggeration to say that this kind of imaging modality had become a gate-keeper for diagnosing coronary artery disease (CAD). Recently, myocardial perfusion CT has been emerging as one option for assessing the hemodynamic significance of CAD [6]. There is a report that shows the transmural extent and severity of myocardial hypoperfusion predicts long-term outcome in NSTE-ACS [7]. Adenosine triphosphate (ATP) stress contrast-enhanced CT can identify both ATP-induced myocardial ischemia and coronary artery stenoses in patients with stable CAD. Moreover, Coronary territory analysis revealed a significant correlation between the severity of coronary stenosis and myocardial blood flow [8]. In a previous report, CT perfusion images were obtained using the prospective ECG-gated dynamic acquisition mode triggered at a phase of 40% RR interval during the administration of contrast medium, and coronary CT angiography images were obtained with prospective ECG gating at a phase of 75% RR interval. ECG-synchronized CT is essential for the accurate evaluation of coronary arteries and perfusion images, but its test procedure is complicated and time-consuming and so it is inappropriate for use in the emergency department.

Conventional contrast-enhanced CT is commonly performed in the emergency department, and it is useful for diagnosing nonischemic cardiovascular life-threatening diseases. On the other hand, there is an opinion that conventional contrast-enhanced CT without ECG synchronization is generally not to be useful for diagnosing CAD. However, it is a fact that there are several reports regarding the effectiveness of contrast-enhanced CT without ECG synchronization for the diagnosis of NSTE-ACS [9, 10]. These papers also say that in many institutions, conventional non-ECG-gated contrast-enhanced CT is performed for patients with acute-onset chest pain to rule out aortic dissection, pulmonary thromboembolism, and other potentially fatal conditions. Our case is exactly one of representative cases in that issue. Because myocardial perfusion in our case was markedly reduced due to severe stenosis of the LCX artery, even conventional contrast-enhanced CT could clearly visualize the reduced myocardial perfusion despite the lack of synchronization with ECG. Thereby, we could make an early and accurate differential diagnosis of the acute chest pain. We reconstructed a volume-rendering CT image afterwards. Moreover, we visualized the area with reduced contrast enhancement (blue area) and the supposed left coronary artery course (yellow dot lines) on the CT image (Fig. 3c) based on the angiographical image (Fig. 3d).

In common, artifacts on CT image such as a consequence of motion, misalignment, and beam hardening sometimes mislead an accurate diagnosis [11]. However, because both of the adjacent spine and aorta which were filled with contrast medium were not relatively close to the area of transmural perfusion defect (Fig. 2), we did not conduct beam hardening correction. As a result, the perfusion defect in our case was thought to be strongly supported by the ischemic change due to coronary stenosis in the LCX artery. Regarding the reason for the discrepancy between transmural perfusion defect on CT image and normal wall motion of the corresponding myocardium on UCG, we think coronary blood flow was significantly disturbed by the tight stenosis in the LCX artery, but its blood supplying area was too small to be detected by the finding of wall motion abnormality on UCG.

We diagnosed a patient with NSTE-ACS in whom contrast-enhanced CT without ECG synchronization was effective for visualization of reduced myocardial perfusion, suggesting IHD. Although the finding in this case is not a universal result, it may be useful to keep in mind that myocardial perfusion can be evaluated by conventional CT in the emergency department when we encounter patients with acute chest symptoms and need to make an early differential diagnosis.

## Data Availability

Data sharing is not applicable to this article as no datasets were generated or analyzed during the current study.

## Abbreviations

ACS: Acute coronary syndrome
NSTE: Non-ST-elevation
ECG: Electrocardiography
CT: Computed tomography
PE: Pulmonary embolism
LCX: Left circumflex
IHD: Ischemic heart disease
CAD: Coronary artery disease
ATP: Adenosine triphosphate
